# The Treadmill Exercise Protects against Dopaminergic Neuron Loss and Brain Oxidative Stress in Parkinsonian Rats

**DOI:** 10.1155/2017/2138169

**Published:** 2017-06-21

**Authors:** Roberta Oliveira da Costa, Carlos Vinicius Jataí Gadelha-Filho, Ayane Edwiges Moura da Costa, Mariana Lima Feitosa, Dayane Pessoa de Araújo, Jalles Dantas de Lucena, Pedro Everson Alexandre de Aquino, Francisco Arnaldo Viana Lima, Kelly Rose Tavares Neves, Glauce Socorro de Barros Viana

**Affiliations:** ^1^Faculty of Medicine of the Federal University of Ceará (UFC), Fortaleza, CE, Brazil; ^2^Faculty of Medicine Estácio of Juazeiro do Norte (Estácio/FMJ), Juazeiro do Norte, CE, Brazil

## Abstract

Parkinson's disease (PD), a progressive neurological pathology, presents motor and nonmotor impairments. The objectives were to support data on exercise benefits to PD. Male Wistar rats were distributed into sham-operated (SO) and 6-OHDA-lesioned, both groups without and with exercise. The animals were subjected to treadmill exercises (14 days), 24 h after the stereotaxic surgery and striatal 6-OHDA injection. Those from no-exercise groups stayed on the treadmill for the same period and, afterwards, were subjected to behavioral tests and euthanized for neurochemical and immunohistochemical assays. The data, analyzed by ANOVA and Tukey post hoc test, were considered significant for *p* < 0.05. The results showed behavioral change improvements in the 6-OHDA group, after the treadmill exercise, evaluated by apomorphine rotational behavior, open field, and rota rod tests. The exercise reduced striatal dopaminergic neuronal loss and decreased the oxidative stress. In addition, significant increases in BDNF contents and in immunoreactive cells to TH and DAT were also observed, in striata of the 6-OHDA group with exercise, relatively to those with no exercise. We conclude that exercise improves behavior and dopaminergic neurotransmission in 6-OHDA-lesioned animals. The increased oxidative stress and decreased BDNF contents were also reversed, emphasizing the importance of exercise for the PD management.

## 1. Introduction

The rising of the older population worldwide is expected to lead to a high prevalence of age-related diseases. Thus, neurodegenerative pathologies like Parkinson's disease (PD) are going to increase, as life expectancy is getting longer, impacting public health-care costs [1]. Exercise is an important part of daily life and, for PD patients, besides being healthy, it is also a vital component for maintaining balance, mobility, and daily activities. In this sense, exercise can be beneficial in symptom management and also possibly slowing disease progression. Furthermore, to reach the pathology recovery, a prominent goal in PD research is finding a neuroprotective treatment that, when applied prior to the onset of the disease, will decrease its risk or severity. One such treatment which has a potential to be a neuroprotective agent in PD is exercise [2].

Neurodegenerative diseases are characterized by a progressive deterioration of brain function, with a consequent and significant decrease in the quality of life of patients and their families. In PD, the progressive loss of dopaminergic neurons in the *substantia nigra pars compacta* leads to motor dysfunction. Thus, evidence [3] indicates that treadmill exercise enhances the survival of dopaminergic neurons in the *substantia nigra*, as well as the fiber projection to the striatum.

It is largely accepted that the exposure to an enriched environment increases neurogenesis in the dentate gyrus of adult rodents [4–7]. Furthermore, evidence has indicated an important role of physical exercise as a potent enhancer of adult hippocampal neurogenesis, pointing it as a potential therapeutic strategy for reducing cognitive decline [8, 9].

Oxidative stress is known to play an important role in the degeneration of dopaminergic neurons in PD. The DA metabolism itself is known to contribute to oxidative stress, as well as mitochondrial dysfunction and the consequent increase in reactive oxygen species (ROS). Evidences from clinical trials have failed to demonstrate any benefit of the oxidative stress for decreasing PD progression. However, recent findings on mechanisms related to PD gene products and neuronal response to stress may provide new targets towards neuroprotection [10].

The objectives of the present work were to add some new information on the role of exercise and oxidative stress in a PD model in rats. We focus on behavioral testing, neurochemical determination of DA and its main metabolites, DOPAC and HVA. In addition, striatal BDNF contents and immunohistochemical assays for tyrosine hydroxylase (TH) and the dopamine transporter (DAT) were also carried out.

## 2. Materials and Methods

### 2.1. Drugs and Reagents

6-hydroxydopamine (6-OHDA), apomorphine, and HPLC standards were from Sigma-Aldrich (St. Louis, MO, USA); ketamine and xylazine were from Konig do Brasil (Santana de Parnaíba, São Paulo, Brazil). The BDNF kit for ELISA was from Abcam (Cambridge, UK), and antibodies for immunohistochemistry assays were from Santa Cruz Biotechnology (Dallas, TX, USA) or Merck Millipore (Darmstadt, Germany). All other reagents were of analytical grade.

### 2.2. Animals

Male Wistar rats (200–250 g) were maintained at a 24 ± 2°C temperature, in a 12 h dark/12 h light cycle, with standard food and water ad libitum. The study was submitted to the Ethical Committee for Animal Experimentation of the Faculty of Medicine of the Federal University of Ceará (Brazil) and was approved under the number 90/2014. All experiments followed the ethical principles established in the Guide for the Care and Use of Laboratory Animals, USA, 2011.

### 2.3. Experimental Procedure for the Treadmill Exercise

**Figure 1:**
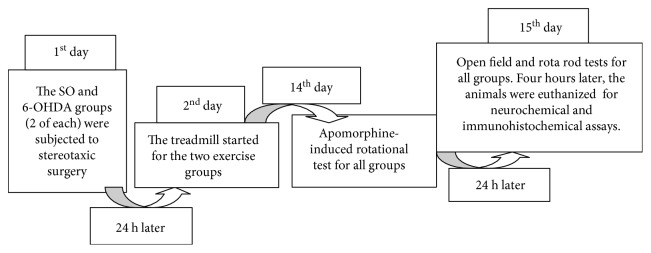
Experimental protocol design.

Male Wistar rats (200–250 g) were distributed into the following groups (6–10 animals each): sham-operated (SO) with or without exercise and 6-OHDA-treated also with or without exercise. The animals were subjected to treadmill physical exercise (30 min, at a speed of 20 cm/s), once a day, for 14 consecutive days. This procedure started 24 h after the stereotaxic surgery and the striatal 6-OHDA injection. For the no-exercise groups, the animals stayed on the treadmill for the same period of time [11, 12]. One hour later, the animals were subjected to the apomorphine-induced rotation test and, in the next day (day 15), to the open field, followed by the rota rod test and, then, euthanized for neurochemical and immunohistochemical assays (see Figure 1).

### 2.4. The 6-OHDA Model of PD and the Experimental Protocol

This is a widely used model of Parkinson disease. The intrastriatal injection of 6-OHDA causes a progressive retrograde and dose-dependent neuronal degeneration in the nigrostriatal pathway [13, 14]. The degree of lesion can be intensified if 6-OHDA is injected into different striatal sites [15]. In the present study, the animals were anesthetized with the association of xylazine (10 mg/kg, i.p.) and ketamine (80 mg/kg, i.p.) and, after head shaving, fixed to the stereotaxic frame by their ear canals. A longitudinal midline incision was made, and the tissues were separated for bregma visualization. Then, a thin hole was performed in the skull over the target area, and a 1 *μ*L solution containing 6 *μ*g 6-OHDA was injected into two different points. The following coordinates were used: 1st point (AP,+0.5; ML,−2.5; DV,+5.0) and 2nd point (AP,−0.9; ML,−3.7; DV,+6.5). The syringe stayed in place for 5 min, to assure the solution diffusion, and, then, the incision was sutured. The sham-operated (SO) animals were subjected to all procedures, except that saline was injected into the two points. Afterwards, the animals returned to their cages for recovery. They were divided into the following groups: SO (with exercise), SO (without exercise), 6-OHDA-lesioned (with exercise), and 6-OHDA-lesioned (without exercise).

### 2.5. Rotational Behavior

The apomorphine induction of rotational (circling) behavior is widely used for assessing the effects of lesions to the dopaminergic system and the success of treatment strategies, in rat models of Parkinson's disease. The number of rotations under apomorphine is related to the extent of dopamine depletion, after the unilateral 6-hydroxydopamine lesion. The contralateral rotations (opposite to the lesioned right side) induced by apomorphine (3 mg/kg, s.c.) were monitored for 1 h. The cause for this apomorphine-induced rotational behavior is related to the unbalance, in the nigrostriatal dopaminergic pathways, between the right (lesioned) and left (unlesioned) brain hemispheres. This asymmetric circling behavior, after the apomorphine administration, is a quantifiable motor deficit and an important paradigm in this model of PD [16, 17]. This test was performed at the 14th day (1 h after the treadmill exercise).

### 2.6. Open Field Test

This test evaluates a stimulant or depressant drug activity and may also indicate an anxiolytic action. The arena was made of wood, whose dimensions were 50 cm × 50 cm × 30 cm (length, width, and height). The floor was divided into 4 quadrants of equal size. At the time of the experiment, the apparatus was illuminated by a red light and was afterwards cleaned with a 70% alcohol solution for avoiding odor interference in the test response. The number of crossings with the four paws from one quadrant to another, for 5 min (parameter for measuring the locomotor spontaneous activity), was determined. This test was performed at the 15th day (24 h after the apomorphine-induced rotational behavior).

### 2.7. Rota Rod Test

The rota rod is a standard test of motor coordination, balance, and fatigue in rodents and is especially sensitive in detecting cerebellar dysfunction. Motor deficits are usually observed in the Parkinson's disease model in rodents. Basically, the animal is placed on a rotating bar, under continuous speed (12 rpm/min), and the time latency/min to fall from the bar is recorded [18]. This test was performed at day 15 (after the open field test).

### 2.8. Neurochemical Determinations of DA, DOPAC and HVA by HPLC

At the 15th day after the treadmill exercise and the apomorphine-induced rotational test, the animals were subjected to the open field and rota rod tests and, 4 h later, euthanized for decapitation and striatal tissue dissection. The striatal contents of DA, DOPAC and HVA were determined by HPLC. Homogenates were prepared in 10% HClO_4_ and centrifuged at 4°C (15,000 rpm, 15 min). The supernatants were filtered, and 20 *μ*L was injected into the HPLC column. For that, an electrochemical detector (model L-ECD-6A, from Shimadzu, Japan) coupled to a column (Shim-Pak CLC-ODS, 25 cm) with a flux of 0.6 mL/min was employed. A mobile phase was prepared with monohydrated citric acid (150 mM), sodium octyl sulfate (67 mM), 2% tetrahydrofuran, and 4% acetonitrile, in deionized water. The mobile phase pH was adjusted to 3.0 with NAOH (10 mM). Monoamines were quantified by comparison with standards, processed the same manner as the samples. The results are expressed as ng/g tissue.

### 2.9. Determination of Nitrite Contents

In this assay, the Griess reagent (1 part 0.1% naphthylethylenediamine dihydrochloride in distilled water plus 1 part 1% sulfanilamide in 5% H_3_PO_4_) indicates the presence of nitrites in the sample. Striatal homogenates (10% in KCl buffer) were centrifuged (12,000 rpm for 10 min), and 100 *μ*L supernatants were added to 100 *μ*L Griess reagent; this mixture stayed on RT for 10 min. The standard NaNO_2_ curve was obtained (in spectrophotometer, at 520 nm) and used for calculating the results expressed as *μ*mol nitrite per g tissue [19].

### 2.10. Determination of Lipid Peroxidation by Thiobarbituric Acid Reactive Substances (TBARS)

Lipid peroxidation expresses oxidative stress induced by ROS reactivity. A largely used method for measuring it is the determination of malondihaldehyde (MDA) in biological samples [20]. Although the lipid peroxidation products are MDA and 4-hydroxy-2-nonenal (4-HNE), MDA is a good biomarker of oxidative stress and an end product of lipid peroxidation [21, 22]. Striatal homogenates (10%) in 1.15% KCl were added (250 *μ*L) to 1 mL 10% TCA, followed by addition of 1 mL 0.6% thiobarbituric acid. After agitation, this mixture was maintained in a water bath (95–100°C) for 15 min. Then, the mixture was cooled on ice and centrifuged (4000 rpm/5 min). The TBARS content was determined in a plate reader, at 540 nm, with results expressed in *μ*mol MDA per g tissue. A standard curve with several MDA concentrations was also performed.

### 2.11. BDNF Measurements in the Rat Striata

Quantification of endogenous brain-derived neurotrophic factor (BDNF) can be performed with an enzyme-linked immunosorbent assay (ELISA). The rat striata were homogenized in PBS (pH 7.4), with the addition of protease inhibitors (Sigma-Aldrich, USA), according to the manufacturer's instructions. The results were expressed as pg/g tissue.

### 2.12. Immunohistochemistry Assays

Brain striatal sections (5 *μ*m) were fixed in 10% buffered formol, for 24 h, followed by a 70% ethanol solution. The sections were embedded into paraffin wax for slice processing on appropriate glass slides. These were placed in the oven at 58°C, for 10 min, followed by deparaffinization in xylol and rehydration in alcohol at decreasing concentrations, and washed in distilled water and PBS (0.1 M sodium phosphate buffer, pH 7.2), for 10 min. The endogenous peroxidase was blocked with a 3% hydrogen peroxide solution, followed by incubation with the appropriate primary anti-antibody, for tyrosine hydroxylase (TH) and dopamine transporter (DAT), and diluted according to the manufacturer's instructions (Santa Cruz or Millipore, USA), for 2 h, at room temperature in a moist chamber. The glass slides were then washed with PBS (3 times, 5 min each) and incubated with the biotinylated secondary antibody, for 1 h, at room temperature. Then, they were washed again in PBS and incubated with streptavidin-peroxidase, for 30 min, at room temperature. After another wash in PBS, they were incubated in 0.1% DAB solution (in 3% hydrogen peroxide). Finally, the glass slides were washed in distilled water and counterstained with Mayer's hematoxylin, washed in tap water, dehydrated in alcohol (at increasing concentrations), diaphanized in xylol, and mounted on Entelan® for optic microscopy examination. The immunostaining intensity was quantified by the Image J software (National Institute of Health, USA), and the results were expressed as relative optical density.

## 3. Statistical Analyses

For statistical analyses, one-way ANOVA, followed by Tukey as the post hoc test, was used for multiple comparisons. Whenever needed, the two-tailed paired or unpaired Student's *t*-tests were used for comparisons between two means. The photomicrograph data were quantified by the Image J software (NIH, USA). Differences were considered significant at *p* < 0.05.

## 4. Results

### 4.1. Apomorphine-Induced Rotation

**Figure 2:**
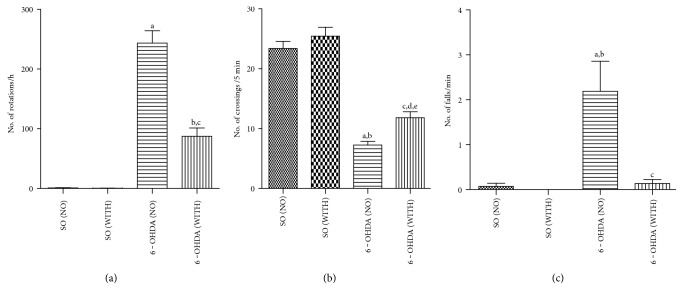
The treadmill exercise significantly reduces behavioral alterations observed in the 6-OHDA group with no exercise. (a) Apomorphine-induced rotational behavior: a versus SO (NO), *q* = 16.25, *p* < 0.001; b versus SO (WITH), *q* = 6.191, *p* < 0.001; c versus 6-OHDA (NO), *q* = 11.69, *p* < 0.001. (b) Open field test: a versus SO (NO), *q* = 15.77, *p* < 0.001; b versus SO (WITH), *q* = 17.19, *p* < 0.001; c versus 6-OHDA (WITH), *q* = 4.382, *p* < 0.05; d versus SO (NO), *q* = 10.63, *p* < 0.001; e versus SO (WITH), *q* = 12.15, *p* < 0.001. (c) Rota rod test: a versus SO (NO), *q* = 5.806, *p* < 0.001; b versus SO (WITH), *q* = 6.111, *p* < 0.001; c versus 6-OHDA (WITH), *q* = 5.739, *p* < 0.001 (one-way ANOVA and Tukey as the post hoc test).

While almost no rotational behavior was seen in SO groups, without or with exercise, 243.6 rotations/h were on the other hand demonstrated in the 6-OHDA-lesioned group without exercise. This value decreased to 87.3 rotations/h (64% reduction) in the 6-OHDA group, after exercise (Figure 2(a)).

### 4.2. Open Field Test

While there was no difference in the number of crossings/5 min in the SO groups, without (23.4 ± 1.210) or with (25.4 ± 1.511) exercise, a significant decrease (7.3 ± 0.586) was seen in the 6-OHDA group without exercise. A partial recovery was observed in the 6-OHDA group with exercise (11.8 ± 1.001) (Figure 2(b)).

### 4.3. Rota Rod Test

There was greater than 2 the number of falls per minute (2.2 ± 0.672) in the 6-OHDA group without exercise. This number felt close to zero in the 6-OHDA group with exercise. No falls were observed in the SO groups without or with exercise (Figure 2(c)).

### 4.4. Measurements of DA and Its Metabolites (DOPAC and HVA) Contents in the Rat-Lesioned Striata

**Figure 3:**
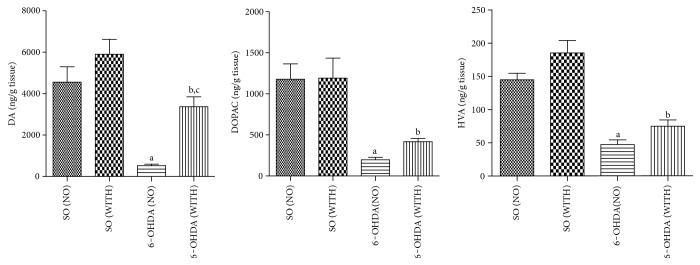
The treadmill exercise decreases the dopaminergic neuron loss of the 6-OHDA-lesioned side, in the 6-OHDA model of Parkinson's disease in rats. DA: a versus SO (NO), *q* = 7.664, *p* < 0.001; b versus SO (WITH), *q* = 5.133, *p* < 0.01; c versus 6-OHDA (NO), *q* = 6.577, *p* < 0.001. DOPAC: a versus SO (NO), *q* = 7.489, *p* < 0.001; b versus SO (WITH), *q* = 5.363, *p* < 0.01. HVA: a versus SO (NO), *q* = 7.513, *p* < 0.001; b versus SO (WITH), *q* = 9.391, *p* < 0.001 (one-way ANOVA and Tukey as the post hoc test).

We showed a decrease of 84% in DA levels, in the 6-OHDA group without exercise, compared with the 6-OHDA group with exercise. This last group showed a reduction of 43% in DA contents, as related to the SO group with exercise. An even higher decrease was observed in the 6-OHDA group without exercise, compared with the SO groups without (89%) and with exercise (91%) (Figure 3(a)). Similarly, smaller decreases (53 and 65%) were seen in DOPAC contents, in the 6-OHDA groups without and with exercise, compared with the 6-OHDA group with exercise and with the SO group in the presence of exercise, respectively (Figure 3(b)). For HVA contents, we also showed smaller decreases (37 and 60%) in the 6-OHDA group, without and with exercise, compared with the 6-OHDA with exercise and with the SO group in the presence of exercise, respectively (Figure 3(c)).

### 4.5. Nitrite Contents and Lipid Peroxidation in Lesioned Striata

**Figure 4:**
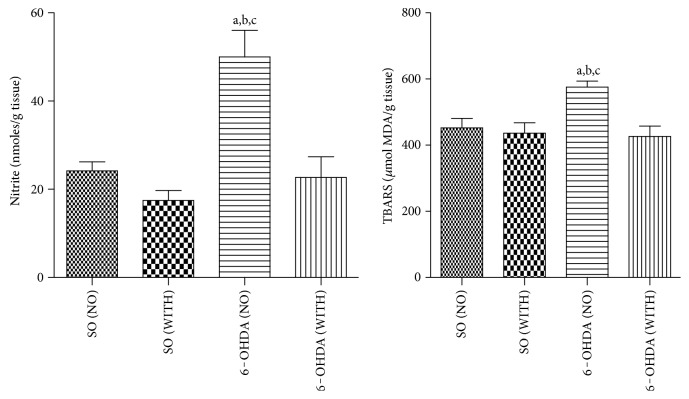
The treadmill exercise reduces nitrite contents and lipid peroxidation of the striatal lesioned side, in the 6-OHDA model of Parkinson's disease in rats. Nitrite: a versus SO (NO), *q* = 4.945, *p* < 0.01; b versus SO (WITH), *q* = 6.609, *p* < 0.001; c versus 6-OHDA (WITH), *q* = 5.555, *p* < 0.01. Lipid peroxidation (TBARS): a versus SO (NO), *q* = 4.209, *p* < 0.05; b versus SO (WITH), *q* = 4.640, *p* < 0.05; c versus 6-OHDA (WITH), *q* = 4.831, *p* < 0.01 (one-way ANOVA and Tukey as the post hoc test).

A significant 55% decrease in nitrite contents was demonstrated in the 6-OHDA group with exercise, as related to the 6-OHDA group without exercise. Similar decreases of 52 and 65% were detected in the 6-OHDA group without exercise, compared with the SO groups without and with exercise, respectively (Figure 4(a)). A 26% decrease in lipid peroxidation was seen in the 6-OHDA group with exercise, as related to the 6-OHDA group without exercise. Similar decreases occurred by comparing the 6-OHDA group without exercise with the SO groups without (22%) and with (24%) exercise (Figure 4(b)).

### 4.6. BDNF Measurements in Striata

**Figure 5:**
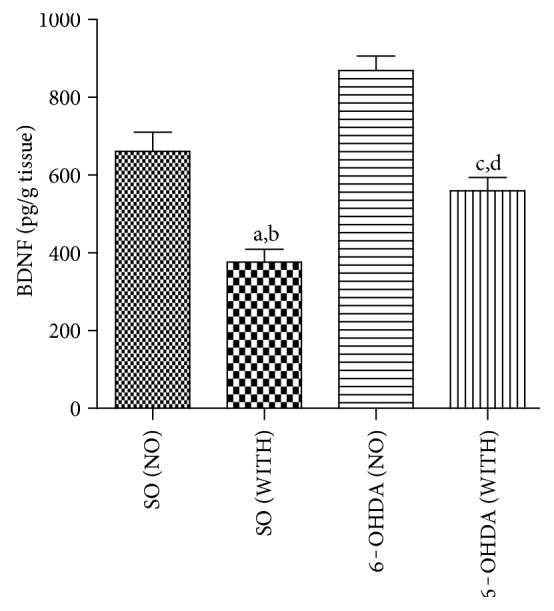
The treadmill exercise significantly increases BDNF contents of the striatal lesioned side, in the 6-OHDA model of Parkinson's disease in rats. a versus SO (NO), *q* = 10.95, *p* < 0.001; b versus SO (WITH), *q* = 14.07, *p* < 0.0.001; c versus SO (WITH), *p* < 8.334; d versus 6-OHDA (NO), *q* = 5.233, *p* < 0.01 (one-way ANOVA and Tukey as the post hoc test).

We showed a reduction of 33% in striatal BDNF levels, in the 6-OHDA group without exercise, compared with the group with exercise. Higher decreases (50 and 57%) were observed in the 6-OHDA group without exercise, in relation to the SO groups without exercise and with exercise, respectively. Interestingly, a 36% reduction in BDNF contents was also seen in the 6-OHDA group with exercise, compared with the SO group with exercise (Figure 5).

### 4.7. Immunohistochemistry Assays for Tyrosine Hydroxylase (TH) and Dopamine Transporter (DAT) in Striata

**Figure 6:**
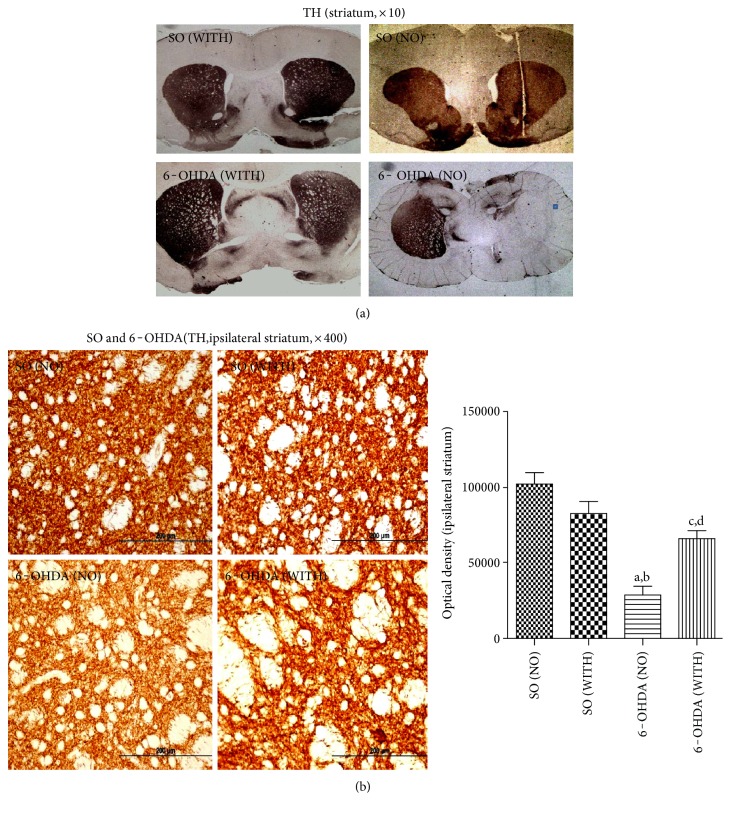
The treadmill exercise increases the immunoreactivity for enzyme tyrosine hydroxylase (TH) of the striatal lesioned side, in the 6-OHDA model of Parkinson's disease in rats. (a) Panoramic images of TH immunostaining in striatal slices (×10 magnification), showing the left (unlesioned) and right (lesioned) sides. (b) Representative photomicrographs of TH immunostaining in striata (×400, scale = 200 *μ*m). a versus SO (NO), *q* = 8.828, *p* < 0.001; b versus SO (WITH), *q* = 6.554; *p* < 0.01; c versus SO (NO), *q* = 4.310, *p* < 0.05; d versus 6-OHDA (NO), *q* = 4.518, *p* < 0.05 (one-way ANOVA and Tukey as the post hoc test).

**Figure 7:**
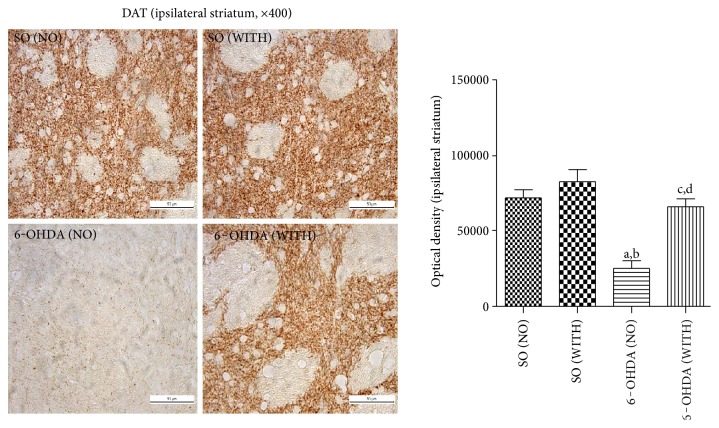
Representative photomicrographs (×400, scale = 50 *μ*m) showing that the treadmill exercise increases the immunoreactivity for the dopamine transporter (DAT) of the striatal lesioned side, in the 6-OHDA model of Parkinson's disease in rats. a versus SO (NO), *q* = 10.54, *p* < 0.001; b versus SO (WITH), *q* = 13.89, *p* < 0.001; c versus SO (WITH), *q* = 6.850, *p* < 0.01; d versus 6-OHDA (NO), *q* = 6.952, *p* < 0.01 (one-way ANOVA and Tukey as the post hoc test).

Reductions of 56 and 71% were demonstrated in TH immunoreactivity in the 6-OHDA group without exercise, compared with the 6-OHDA group with exercise and with the SO group without exercise (Figure 6). Interestingly, no significant difference was seen between the 6-OHDA with exercise and the SO also with exercise groups. Similarly, 60 and 70% reductions were demonstrated in DAT immunoreactivity in the 6-OHDA group without exercise, as related to the 6-OHDA group with exercise and to the SO group without exercise, respectively. A decrease of only 37% was observed in the 6-OHDA group with exercise, in relation to the SO group with exercise (Figure 7).

## 5. Discussion

Parkinson's disease (PD) is a debilitating pathology associated with dopaminergic neuron loss in the *substantia nigra pars compacta.* PD is characterized by motor symptoms, as bradykinesia, tremor, muscular rigidity and postural instability, as well as nonmotor symptoms, as depression, anxiety, and cognitive deficits, among others. Although Levodopa is the gold standard pharmacological treatment, it is only symptomatic and does not reduce the disease progression. Oral Levodopa has been widely used for over 40 years, often in combination with a dopa-decarboxylase inhibitor [23, 24]. However, after an initial beneficial period, Levodopa shows several limitations from nonmotor and mainly motor origins, as dyskinesias [25].

Although the human brain shrinks with advancing age, it is also capable of remarkable plasticity. Evidence [2] indicates that physical activity is a promising intervention, influencing the endogenous pharmacology of the brain and enhancing cognitive, as well as emotional function in late life. Animal studies also support the benefits of exercise-induced neuroplasticity of corticostriatal circuits that are profoundly affected in Parkinson's disease [26, 27]. It is, then, reasonable to state that physical activity is becoming a very important instrument for PD patients, since, by increasing the overall physical capacity, it reduces the risk of falls and injuries and improves the quality of life and cognitive function [28, 29].

In the present work, we showed that the treadmill exercise decreases the rotational behavior induced by apomorphine, in 6-OHDA-lesioned rats. The motor coordination, as evaluated by the open field and rota rod tests, was also improved. Others [30] suggest that exercise is neuroprotective, improving deteriorated motor function in a PD model similar to ours, but using female rats instead. Furthermore, parkinsonian animal models indicate that exercise protects the brain from dopaminergic neurotoxins, possibly mediated by brain neurotrophic factors and neuroplasticity [31].

Our treadmill exercise protocol in parkinsonian rats revealed a decreased dopaminergic loss in the striatum, in the group with exercise, supporting the neuroprotective effect of exercise. Thus, not only DA but also its metabolites, DOPAC and HVA, showed a lower reduction in their striatal contents in the presence of exercise. A similar result was demonstrated by others, pointing out that exercise recovers Parkinson's disease-induced dopaminergic neuron loss [32].

Oxidative stress seems to be involved in the pathogenesis of PD, and exercise can increase endogenous antioxidant protection and decrease ROS production [33]. Oxidative stress is associated with other components of the degenerative process, such as mitochondrial dysfunction, excitotoxicity, nitric oxide toxicity, and inflammation [34]. The impaired mitochondrial function, as demonstrated in PD patients, is likely to increase the oxidative stress, rendering cells more vulnerable to this and other processes, as excitotoxicity [35]. Increasing evidence indicates that oxidative stress predisposes cells to damage in DNA, proteins, and lipids, common factors involved in the pathogenesis of neurodegenerative diseases, as PD [36]. The challenge is to minimize the elevated and uncontrolled oxidative stress.

Furthermore, evidence indicates that prolonged or short-duration high intensity exercises arise increased radical production in active skeletal muscles, resulting in the production of oxidized lipids and proteins [37, 38]. On the other hand, regular exercise with moderate intensity and duration has a wide range of beneficial effects on the body and may reduce the incidence of neurodegenerative diseases, as PD, by enhancing the concentration of neurotrophins and by modulation of redox homeostasis [39].

We showed that the treadmill exercise significantly decreases nitrite levels and lipid peroxidation, in rats subjected to 6-OHDA lesion in the striatum. Previously [40], an increased brain oxidative stress has been suggested as a predictor of cognitive impairment in Alzheimer's disease, a neurodegenerative disorder as Parkinson's disease, what strengthens the importance of decreasing the brain oxidative stress under these conditions.

Furthermore, we demonstrated a decrease in BDNF contents in the 6-OHDA-lesioned striatum without exercise, in relation to this group with exercise. The decrease was even higher when the lesioned group without exercise was compared to the SO group with exercise. Interestingly, a similar pattern was also observed in other brain areas, such as the prefrontal cortex and hippocampus (data not shown). Thus, exercise increased BDNF concentrations in the rat brain and this neutrophic factor is certainly involved with the exercise neuroprotective effect.

Evidences [41] indicate that the hippocampus decreases in size with aging, leading to impaired memory and increased risk for dementia, but exercise increases hippocampal size. These authors demonstrated that increased hippocampal volume is associated with greater serum levels of BDNF. The brain-derived neurotrophic factor (BDNF) is implicated in the regulation of neuronal survival, cell differentiation, and synaptic plasticity, and its reduced expression in the *substantia nigra* seems to be associated with dopaminergic neurons loss, in PD patients [42]. Furthermore, it has been demonstrated that exercise protects dopaminergic neurons of the *substantia nigra pars compacta* from neurotoxicity, and a mechanism proposed to account for this neuroprotection is the upregulation of neurotrophic factors, as BDNF [43]. Decreased BDNF levels are associated with cognitive deficits in PD patients, and motor rehabilitation, besides improving PD symptoms, also increases BDNF levels [44, 45], supporting our findings.

BDNF exhibits a potent effect on the survival and morphology of dopaminergic neurons, and its loss could contribute to neuron death in PD [46]. BDNF is a small protein widely expressed in the adult mammalian brain which promotes survival of neurons in neurodegenerative diseases, including PD [47]. Due to its colocalization with dopaminergic neurons and its role in cognition, BDNF may exhibit both neuroprotective and neuromodulator roles in PD [48]. Evidence indicates that exercise improves cognition and mood, and preliminary data suggest that BDNF may mediate these effects [49]. Interestingly, these authors also demonstrated moderate and higher BDNF increases, respectively, following a single or a session of exercise.

The striatal injection of 6-OHDA was shown to lead to a massive disappearance of TH immunoreactive terminals, in a defined area within the striatum, surrounding the injection site [50]. TH is the rate-limiting enzyme for brain catecholamine synthesis, and reduction of TH expression results in diminished DA synthesis, leading to PD, what makes TH essential in the pathogenesis of PD [51]. Thus, PD can be considered as a TH-deficiency syndrome of the striatum [52]. Recent data [53] demonstrated that exercise mediates increase in nigral TH expression, in aging rats. We showed that the treadmill exercise of 6-OHDA-lesioned animals partly recovered the decrease in TH immunoreactivity, in the rat striatum. Considering the great importance of TH activity in PD, a logical and efficient therapeutic strategy for its treatment could be based on correcting TH deficiency.

We also demonstrated a decrease in DAT immunoreactivity in the 6-OHDA-lesioned animals without exercise, and this was also reduced by the treadmill exercise. The dopamine transporter (DAT) is a transmembrane protein, responsible for the reuptake of DA from the synaptic cleft and also for the termination of dopaminergic transmission. Although it is usually accepted that parkinsonian symptoms develop when approximately 70–80% of DA neurons are lost, imaging studies have shown that a loss around 50% of DA terminals is required for the onset of symptoms in PD patients [54]. There is an exponential decline of DAT, as the disease progresses, that can be revealed by imaging technics [55], making DAT a marker for distinguishing PD patients from healthy individuals [56].

We conclude that the treadmill exercise really improves the behavioral, neurochemical, and immunohistochemical changes observed in the 6-OHDA-lesioned group without exercise. Several factors seem to be responsible for that, as the decrease in oxidative stress, reduced lipid peroxidation and mainly upregulation of BDNF in the lesioned striata of the 6-OHDA group with exercise. Thus, exercise may offer protection for PD patients, for motor and nonmotor impairments, and should be recommended as part of the routine management and neurorehabilitation of this disorder. The present results should stimulate translational studies, in order to clarify what type and duration of exercise could best improve the quality of life of PD patients.
