# Corrigendum to “The Treadmill Exercise Protects against Dopaminergic Neuron Loss and Brain Oxidative Stress in Parkinsonian Rats”

**DOI:** 10.1155/2018/9650361

**Published:** 2018-05-02

**Authors:** Roberta Oliveira da Costa, Carlos Vinicius Jataí Gadelha-Filho, Ayane Edwiges Moura da Costa, Mariana Lima Feitosa, Dayane Pessoa de Araújo, Jalles Dantas de Lucena, Pedro Everson Alexandre de Aquino, Francisco Arnaldo Viana Lima, Kelly Rose Tavares Neves, Glauce Socorro de Barros Viana

**Affiliations:** ^1^Faculty of Medicine of the Federal University of Ceará (UFC), Fortaleza, CE, Brazil; ^2^Faculty of Medicine Estácio of Juazeiro do Norte (Estácio/FMJ), Juazeiro do Norte, CE, Brazil

In the article titled “The Treadmill Exercise Protects against Dopaminergic Neuron Loss and Brain Oxidative Stress in Parkinsonian Rats” [1], there were errors in Figure 5 and its legend. The corrected figure and legend are shown below.

## Figures and Tables

**Figure 1:**
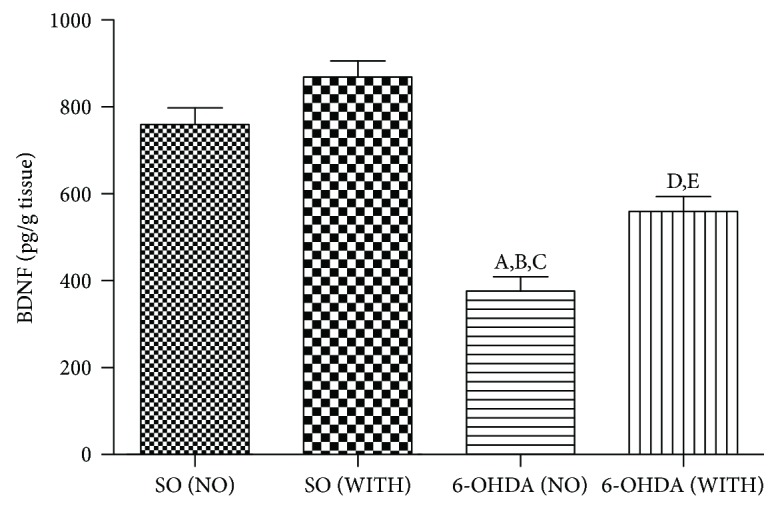
The treadmill exercise significantly increases BDNF contents of the striatal lesioned side, in the 6-OHDA model of Parkinson's disease in rats. a. versus SO (NO), *q* = 10.95, *p* < 0.001; b. versus SO (WITH), *q* = 14.07, *p* < 0.001; c. versus 6-OHDA (WITH), *q* = 5.233, *p* < 0.01; d. versus SO (NO), *q* = 5.388, *p* < 0.01; e. versus SO (WITH), *q* = 8.334, *p* < 0.001 (one-way ANOVA and Tukey as the post hoc test).
